# A case of *Beauveria bassiana* keratitis confirmed by internal transcribed spacer and LSU rDNA D1–D2 sequencing

**DOI:** 10.1002/nmi2.30

**Published:** 2014-05-20

**Authors:** M Ligozzi, L Maccacaro, M Passilongo, E Pedrotti, G Marchini, R Koncan, G Cornaglia, A R Centonze, G Lo Cascio

**Affiliations:** 1 Microbiology and Virology Unit, Department of Pathology and Diagnostic, University of VeronaVerona, Italy; 2 Eye Clinic, Department of Neurological and Visual Sciences, University of VeronaVerona, Italy

**Keywords:** *Beauveria bassiana*, keratitis, molecular identification

## Abstract

We describe a case of fungal keratitis due to *Beauveria bassiana* in a farmer with Fuchs' dystrophy, treated with amphotericin B. Surgery with penetrating keratoplasty was necessary to resolve the lesions. Susceptibility testing and molecular sequencing permitted the identification and treatment of this rare aetiological agent of invasive fungal disease.

## Case Report

**Figure 1:**
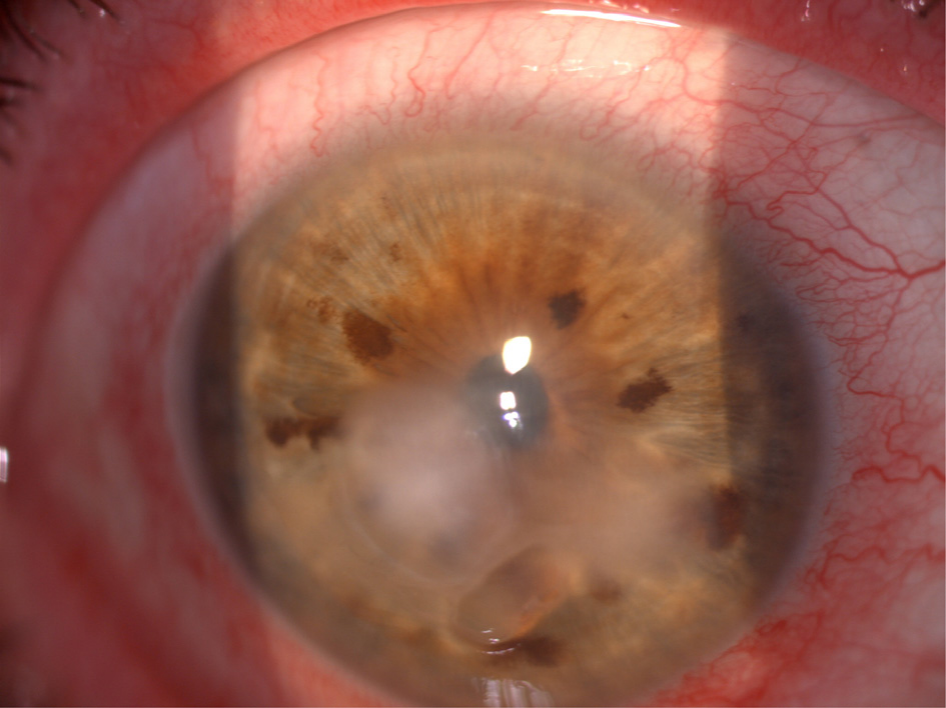
Perikeratic hyperaemia with stromal keratitis.

In early December 2012, a 76-year-old woman affected by chronic obstructive pulmonary disease, chronic atrial fibrillation, arterial hypertension, rheumatoid arthritis, hypothyroidism, osteoporosis and Fuchs' dystrophy presented to the hospital for corneal oedema with a white infiltrated ulcer in the right eye (Fig.1), without previous history of trauma.

The woman lived in a rural area near Mantova, in northern Italy. She bred cattle, but she denied any contact with pesticides.

The patient had already received a corneal endothelial transplant in the left eye in June 2012 for Fuchs' dystrophy, while a perforating keratoplasty was already planned in the right eye for a previous central leucoma and Fuchs' dystrophy in January 2013. The patient's right eye was receiving local chronic therapy with dexamethasone 0.2% eye drops three times a day and ganciclovir gel three times a day.

The patient had already been hospitalized twice for bacterial infection in the right eye (July and December 2011) with positive culture for *Serratia marcescens*; she also suffered with recurrent herpes simplex keratitis in this eye.

Her visual acuity was 1.30 LogMAR (logarithm of the minimum angle of resolution) on the right and 0.07 LogMAR on the left.

**Figure 2:**
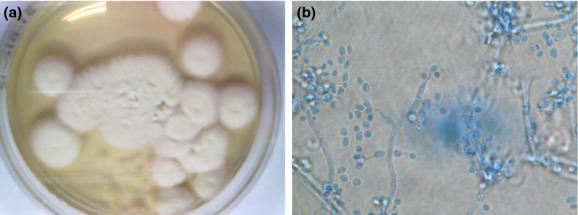
Macroscopic and microscopic appearance of the isolates. (a) Rapid growing, white and densely woolly fungus with a white reverse. (b) Microscopic examination of *Beauveria bassiana* show cell with globose bases and extended, denticulate rachis and the conidia is globose in shape (<3.5 μm diameter). The spore balls representing dense clusters of large numbers of conidiogenous cells and conidia (lactophenol cotton blue stain, ×400).

Two corneal scrapings were collected for conventional microscopic and cultural diagnostic procedures. Microscopic examination of corneal scrapings with calcofluor white fluorescent staining did not show the presence of fungal elements. The culture media used were blood agar, MacConkey's agar, chocolate agar and Sabouraud's dextrose agar (SDA). Corneal scrapings were plated into SDA (Oxoid, Basingstoke, UK) and incubated at 25, 30 and 37°C. One week later, her visual acuity was stable and the stromal keratitis was unchanged; the cultures were positive for *Staphylococcus epidermidis* and for a mould compatible with *Beauveria* spp. 1. Colonies were fast growing, reaching diameters of 4.5 cm in 13 days at 30°C on potato dextrose agar (Difco Laboratories, Detroit, MI, USA). They were densely cottony to flocculent, with droplets of exudate on the surface, yellowish white, raised and dome shaped. For identification of fungal species grown in SDA, fragments of culture were placed onto glass slides for staining with lactophenol cotton blue. Conidiogenous cells occurred in sporodochial clusters and were slightly swollen at the base and narrowed at the tip to form a zigzag rachis. Conidia were oval to subglobose and apiculate, and measured 2 to 3 μm long and 1.5 to 2 μm wide (Fig.2). The difficulties in identifying isolates of *Beauveria* based solely on morphological characteristics have led researchers to consider the use of molecular tools. Total genomic DNA was extracted from mycelia of the fungus grown on potato dextrose agar medium using the silica beads method. In brief, fungal mycelium was harvested from culture plates and was added to 1.5 mL microcentrifuge tubes. Mycelium material was suspended in 200 μL of a bead beating solution containing TE buffer (Tris–HCl 10 mM (pH 8), EDTA 1 mM). Approximately 100 mg of mixed diameter (425–600 μm) glass beads (Sigma Aldrich, St Louis, MO, USA) for crushing of cell walls were also added. The tubes were then placed into a Turbo Mix TM adapter (Scientific Industries Inc., VWR International, Milan, Italy) attachment for a Vortex Genie 2 (Fisher Bioblock Scientific, Fischer Scientific SAS, Ilikirh Cedex, France) and homogenized for 10 min at maximum speed. Then, the tubes were centrifuged for 10 min at 11 000 ***g***. After centrifugation, the supernatants were decanted to a sterile 1.5 mL microcentrifuge tube, and heated to 95°C for 10 min. The DNA obtained by this procedure was suitable in less than an hour for PCR amplification. Internal transcribed spacer (ITS) primers ITS1 and ITS4 were used to amplify a ribosomal DNA (rDNA) ITS region 2. Primers NL1 and NL4 were used to amplify the D1–D2 region of the large-subunit (LSU) rDNA gene 3. Both PCR-amplified fragments were purified using the QIAquick PCR purification kit, according to the manufacturer's instructions (Qiagen, Hilden, Germany). The ITS and D1/D2 regions of the 26S ribosomal DNA was sequenced in both directions using Big Dye Terminator v. 3.1 (Applied Biosystems, Foster City, CA, USA) in an ABI Prism 3100 sequencer (Applied Biosystems). Amplified ITS sequences were compared with the GenBank Nucleotide Database (http://www.ncbi.nlm.nih.gov) using the algorithm Blast N 4. The sequence of the nuclear ribosomal ITS region from the case isolate was deposited in GenBank with the accession number for ITS region HF675188 and for D1/D2 region HF675189.

The *in vitro* activities of amphotericin B, fluconazole, itraconazole, voriconazole, posaconazole, anidulafungin, caspofungin, micafungin and flucytosine were determined by a broth microdilution method according to the standardized procedure for antifungal susceptibility testing proposed by the Clinical and Laboratory Standards Institute (CLSI) document M38-A2 5. Readings were taken at 24 and 48 h. MIC results were 2 mg/L for amphotericin B, >64 mg/L for fluconazole, 0.25 mg/L for itraconazole and voriconazole, and 0.06 mg/L for posaconazole. The minimum effective concentrations of echinocandins were 4 mg/L for anidulafungin and caspofungin, and >8 mg/L for micafungin.

The patient was given a topical galenic therapy with ceftazidime 0.9% eye drops six times a day, vancomycin 5% eye drops six times a day and amphotericin B 0.15 % eye drops six times a day 6–8.

Although the strain isolates showed susceptibility only for voriconazole and posaconazole, we continued the therapy with amphotericin B and vancomycin eye drops six times a day, because neither voriconazole or posaconazole galenic eye drops were available. Ceftazidime administration was discontinued.

A week later the keratitis had improved, the stromal infiltrate appeared to be smaller, and the anterior chamber was always soft. After 30 days the corneal infiltrate was clearer than before and the ulcer had resolved, so we performed the planned perforating keratoplasty with phacoemulsification and implant of an intraocular artificial lens in the right eye 9–11.

At the 20th day the keratoplasty was clear without signs of infection. The patient presented best corrected visual acuity of 0.52 LogMar, intraocular pressure of 18 mmHg, and her therapy was netilmicin 0.3% + dexamethasone 0.1% eye drops six times a day, amphotericin B 0.15% eye drops six times a day and acyclovir 400 mg orally three times a day.

Mycotic keratitis is an important ophthalmic problem in all parts of the world; it is a major cause of visual loss especially in developing countries, where a large part of the population works in agriculture. Risk factors include the widespread use of broad-spectrum antibiotics and steroids, the frequent and sometimes prolonged use of contact lenses, and the growing number of corneal surgeries. Ocular infections caused by *Beauveria bassiana* are extremely rare in humans and there are few reports in the literature 7,12–14. *Beauveria bassiana* is actually a fungus that grows in soils naturally all over the world. It acts as a parasite on many different insect species causing white muscardine disease. It is used in agriculture in pest control as a natural biological insecticide 15–17.

Identification of *Beauveria* species by conventional mycological methods is often difficult. Molecular techniques such as ITS rDNA and LSU rDNA D1–D2 sequencing have become reliable and are highly suitable tools for rapid identification of human pathological moulds 18–20.

In this report, the patient with a fungal corneal infection had agricultural experience. Although phenotypical characterization of *Beauveria* species was controversial and not sufficient to differentiate among strains 21, a further molecular study revealed almost 100% identity with sequences deposited in the GenBank Nucleotide Database, which were able to identify the species as *B. bassiana*. In particular the rapid DNA extraction method allowed a rapid sequencing result.

This isolate displayed *in vitro* resistance to most antifungal agents tested, including amphotericin B, which was empirically used in topical formulation. The *in vivo* response shows a stabilization of the corneal lesion, allowing amphotericin B to inhibit worsening of the disease as seen in previous reports 22.

The underlining disease, Fuchs' dystrophy, mandated a surgical option as the only way of clearing the focus of infection 13. As lamellar keratoplasty would have been ineffective in our patient; we chose to perform a penetrating keratoplasty (PK) with topical use of amphotericin B 7. Although susceptibility testing has been standardized for the most common aetiological agents of invasive fungal disease, like *Aspergillus* and conidia-forming moulds, the clinical significance of this result is not clear and meaningful data are difficult to generate. This is particularly true for amphotericin B, whose *in vitro* MIC values are frequently misleading. The demand for fungal susceptibility testing, created in part by the increase in serious fungal infections and the availability of alternative therapeutic regimens, strongly require more scientific efforts to correlate MIC results with clinical outcome.
